# Automated characterisation of microglia in ageing mice using image processing and supervised machine learning algorithms

**DOI:** 10.1038/s41598-022-05815-6

**Published:** 2022-02-02

**Authors:** Soyoung Choi, Daniel Hill, Li Guo, Richard Nicholas, Dimitrios Papadopoulos, Maria Francesca Cordeiro

**Affiliations:** 1 UCL Institute of Ophthalmology, London, EC1V 9EL UK; 2 Division of Brain Sciences, Department of Medicine, Imperial College, London, UK; 3 Population Data Science, Swansea University Medical School, Swansea, SA2 8PP UK; 4 Laboratory of Molecular Genetics, Hellenic Pasteur Institute, 11521 Athens, Greece; 5 School of Medicine, European University Cyprus, 2414 Nicosia, Cyprus; 6 Imperial College Ophthalmology Research Group, Imperial College London, London, UK

**Keywords:** Microglial cells, Senescence

## Abstract

The resident macrophages of the central nervous system, microglia, are becoming increasingly implicated as active participants in neuropathology and ageing. Their diverse and changeable morphology is tightly linked with functions they perform, enabling assessment of their activity through image analysis. To better understand the contributions of microglia in health, senescence, and disease, it is necessary to measure morphology with both speed and reliability. A machine learning approach was developed to facilitate automatic classification of images of retinal microglial cells as one of five morphotypes, using a support vector machine (SVM). The area under the receiver operating characteristic curve for this SVM was between 0.99 and 1, indicating strong performance. The densities of the different microglial morphologies were automatically assessed (using the SVM) within wholemount retinal images. Retinas used in the study were sourced from 28 healthy C57/BL6 mice split over three age points (2, 6, and 28-months). The prevalence of ‘activated’ microglial morphology was significantly higher at 6- and 28-months compared to 2-months (p < .05 and p < .01 respectively), and ‘rod’ significantly higher at 6-months than 28-months (*p* < 0.01). The results of the present study propose a robust cell classification SVM, and further evidence of the dynamic role microglia play in ageing.

## Introduction

**Figure 1:**
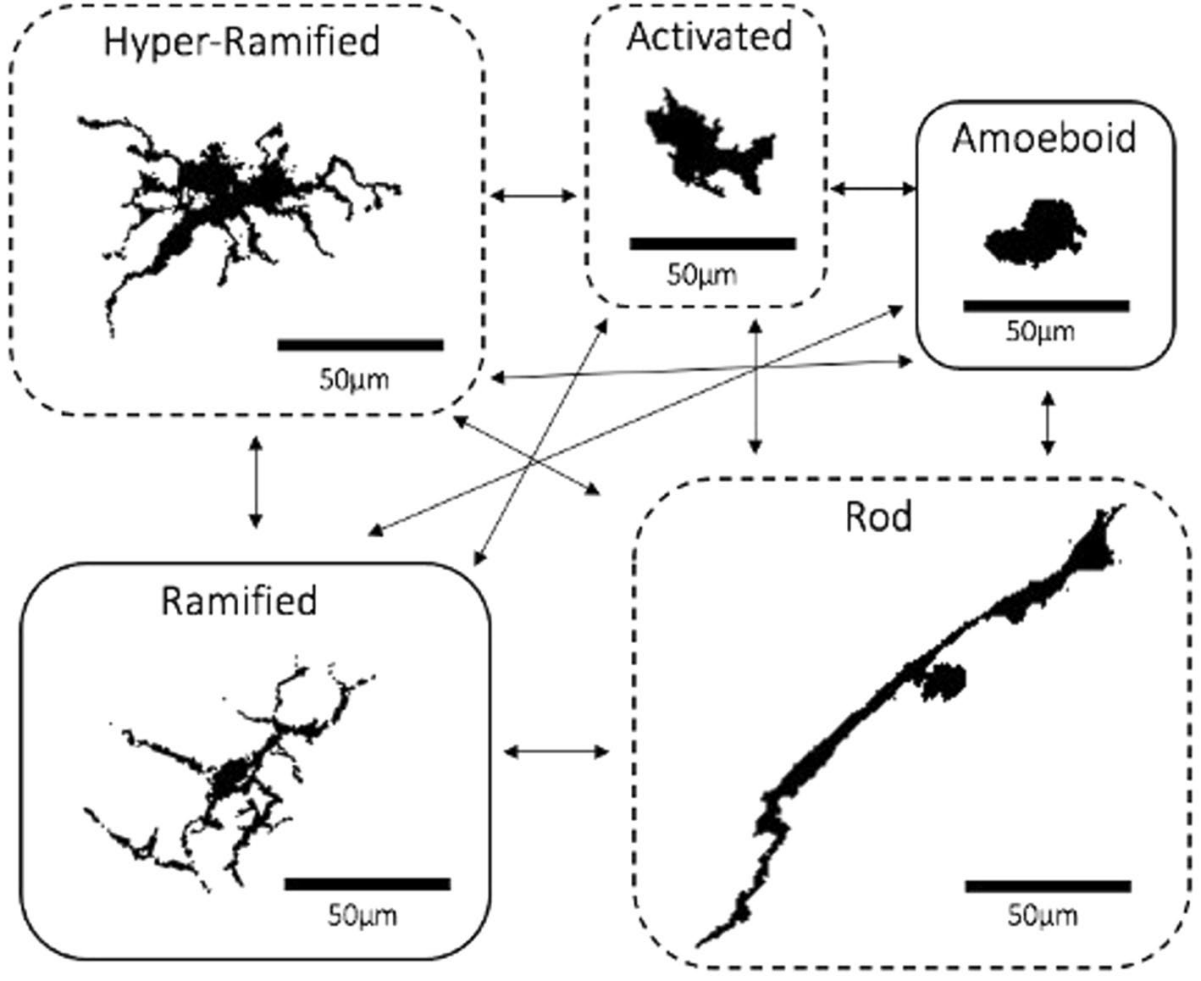
Hypothetical Morphotypes of Microglia. Each microglia morphotype was taken from flat-mounted retinas of C57BL6 mice which were stained with Iba-1 using the staining method in^16^. Diagram taken from^8^. The morphotypes in dashed bubbles (hyper-ramified, activated and rod) may be considered the intermediate states between the ramified and amoeboid.

Microglia are the resident macrophages that make up 5–10% of the cells in the central nervous system (CNS) including the retina^1,2^. Microglia typically reside in the inner and outer plexiform layers of the retina^3^. In contrast to initially being described as quiescent cells surveying their microenvironment, microglia are now understood to possess dynamic characteristics and play a more active role within the CNS^2,4^. The dynamic activity of microglia, in response to various stimuli and insults, may be facilitated by altering their morphology into five distinctive shapes or ‘morphotypes’^2,5–8^. The resting ramified morphotype typically have a small and round cell body connected to long and thin processes used to monitor their microenvironment (Fig. 1)^3,4,9^. Microglia may transform into the ‘hyper-ramified’ type when they initially detect a stimulus^10,11^. The ‘hyper-ramified’ cell body is large, irregular and lobular, attached to a higher number of processes that may also be thicker than those of ramified cells (Fig. 1)^10,11^. Stress-related stimuli have been associated with ‘activated’ microglia which also have irregular bodies but with fewer and shorter processes^3,10,12^. Additionally, the ‘rod’ type with a narrow-elongated body with bipolar processes may be seen in disease and ageing (Fig. 1)^4,13–15^. Finally, the ‘amoeboid’ microglia appear with a large cell body that has few or no processes, often associated with phagocytic functions (Fig. 1)^4,8,16^. It is believed that microglia may quickly shift between these morphotypes in response to changes in the CNS microenvironment and exert neuromodulatory functions by controlling factors, cytokines, and chemokines which are secreted^2,5–8^. As a result, characterising retinal microglia in the context of age-related retinal and neurological diseases has become of a popular field of interest^15,17,18^. Despite this, there are few investigations to date on the effects of ageing on the characteristics of microglia^19,20^.

There are other methods to categorise and characterise microglia such as damage associated microglia and the M1/M2a-c sub-types of microglia^8^. These can be defined using both morphological and genetic profiling by observing the expression levels of certain proteins including cytokines^5,8,21,22^. Whilst genetic profiling may elucidate functional associations of microglia types, it may require using several markers simultaneously, resulting in complex and costly investigations. This would be a result of having to find the optimal combination of all primary and secondary antibodies which, with all required reagents would surmount to a large expense. This study therefore investigates the morphological differences observed from images of retinal microglia, and made the changes visualisable using a single fluorescent marker, ionized calcium-binding adapter molecule 1 (Iba-1), which is one of the most commonly used labels of microglia^23,24^.

Although there have been a few studies which investigate retinal microglia characteristics with ageing, these have used manual observations looking at cross-sections of the retina or specific sections of a whole-mounted retina^19,20^. Recent advances in technology have enabled the development of automated methods to investigate densitometric, morphometric, and spatial parameters using freely available software such as ImageJ^16,25^. Additionally, support vector machines (SVMs) have become increasingly used to classify medical imaging data^26–28^. Supervised SVMs may be trained with training data that are categorised into each of the classes that the testing data will need to be grouped into. It can then extract all the unique features that define a class by establishing the optimal hyperplane that distinguishes between all the data of one category from those of all the others^8,29^. Using automated methods to extract morphological characteristic data to train SVMs can classify microglia in the ageing retina as a whole organ. This may provide an objective, accurate and representative method to monitor the microglial dynamics through the ageing process.

In the present study, we have developed automated algorithms to identify any differences in the density of total retinal microglia and percentages of each morphotype per retina between three age groups of healthy mice. Additionally, any differences in the retinal perivascular regions have also been investigated^8^.

## Results

### CBCS and SVM-C development and validation

**Figure 2:**
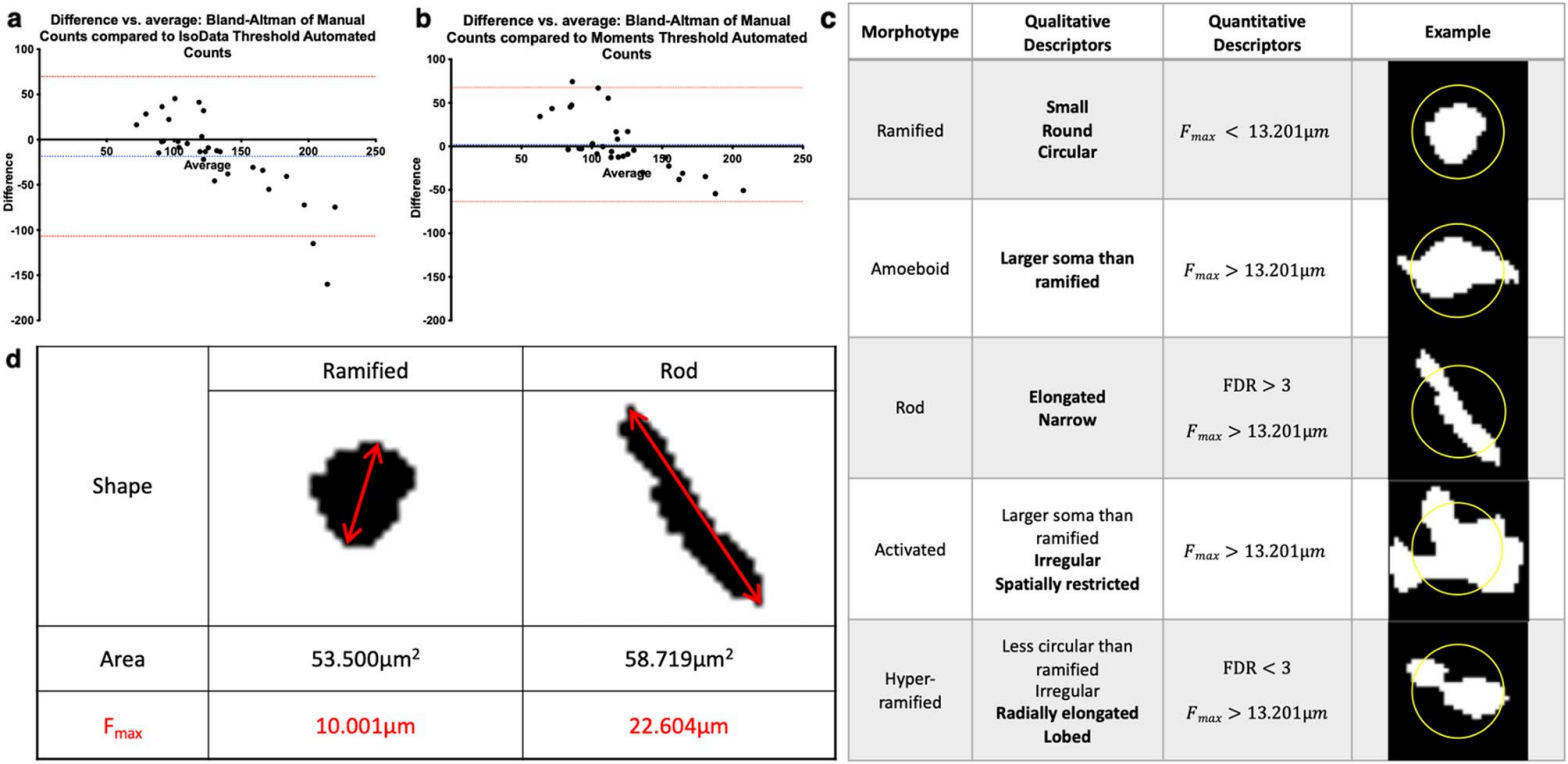
Qualitative and Quantitative Descriptions of Microglia Morphotypes. The Bland-Altmann graphs show the difference between either IsoData (**a**) or Moments (**b**) CBCS cell counts and the manual counts on the y axis, against the average of IsoData or Moments and the manual counts. The dotted lines in blue show the average of differences. That in red show the average $\pm$ 1.96 Standard Deviation. For (**c**), literature references^4,10,12,16,30–36^ were used to deduce qualitative descriptors of the cell bodies of each microglia morphotype. The unique descriptors per morphotype are in bold. Literature reference^10^ and own data were used to deduce the quantitative descriptors of the cell bodies of each microglia morphotype. The examples are the binarized images obtained from the AS to show the cell body images per microglia morphotype. The yellow circle has a diameter of 13.201 μm. ${F}_{max}$ = Feret’s Maximum Diameter; *FDR* Feret’s Diameter Ratio. (**d**) shows the shape of ramified and rod cell bodies to explain how ${F}_{max}$ may be an easier quantitative parameter to distinguish between both cell body morphotypes than area. The areas of the two shapes are very similar (53.500 vs 58.719μm^2^) whilst the ${F}_{max}$ of the ramified is less than half of that of the rod cell body (10.0001 vs 22.604 μm).

The Cell Body Counting Script CBCS was developed and optimised by examining different sub-types of image processing functions such as the thresholding. The CBCS was run on retinal images (n = 30) with either the ‘Moments’ or ‘IsoData’ threshold then both datasets were compared with the results from the average of three manual observers. The Blandt Altmann analyses showed that the ‘Moments’ threshold had narrower limits of agreement (− 63.29 and 67.58) compared to that of the ‘Isodata’ (− 106.9 to 69.69) (Fig. 2a,b).

**Figure 3:**
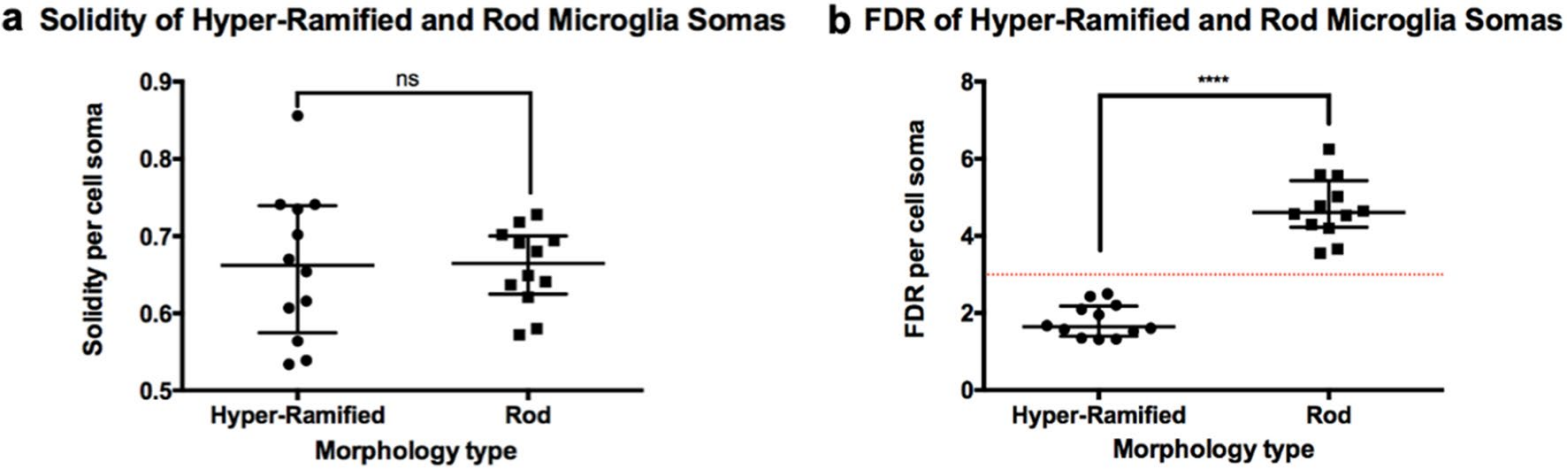
Cell solidity and FDR: Hyper-ramified and Rod microglia. Graphs showing no significant difference in solidity between hyper-ramified and rod microglia (**a**) whilst there was a significant difference in FDR (**b**) (****p < 0.0001, two-tailed Mann–Whitney test).

Although there were some overlapping descriptions, each morphotype was assigned a unique qualitative description based on the available literature (Fig. 2c)^4,10,12,16,30–36^. These descriptions were used to identify a starting point for cells of each morphotype by correlating to the segmented cell body images (Fig. 2c). From this, it was deduced that the ramified cell body would have the smallest F_max_, then the amoeboid being the next largest whilst the other non-ramified morphotypes would have similar or greater values (Fig. 2c). Therefore, the F_max_ of all non-ramified morphotypes were estimated as an average from measurements given in literature and our own data (Fig. 2c)^10^. Other parameters given by the CBCS were investigated to seek for additional quantitative descriptors that could distinguish hyper-ramified from rod. Parameters such as solidity did not show significant differences (Fig. 3a) whilst, the FDR showed a significant difference (p < 0.0001) with a clear cut-off point of 3 (Fig. 3b). These additional quantitative descriptors where then used to further refine the initially identified cells’ data per morphotype. Finally, each identified cell per morphotype were cross referenced again with their corresponding segmented cell body image.

**Figure 4:**
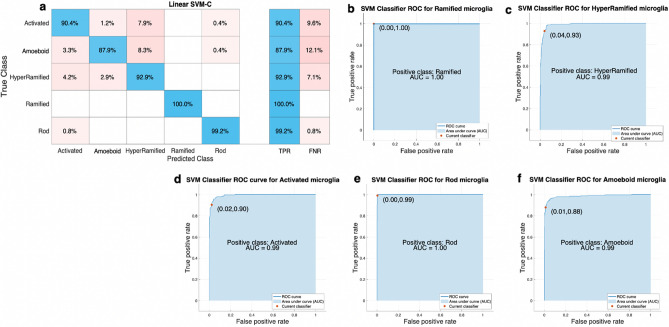
SVM Classifier Confusion Matrix and ROC Curves. (**a**) shows a confusion matrix of the trained SVM Classifier. The rows display the ‘True Class’ and the columns the ‘Predicted Class’. The diagonal cells in blue display the correctly classified observations. The other pink shaded cells display the incorrectly classified observations. *TPR* True Positive Rate, *FNR* False Negative Rate. (**b**–**f**) show the receiver operating characteristic (ROC) curves of the TPR against the false positive rate (FPR) for ramified (**b**), hyper-ramified (**c**), activated (**d**), rod (**e**) and amoeboid (**f**) microglia.

The resulting data (n = 240 per morphotype) were then used to train the linear SVM-C. The true positive rates (TPR) and true negative rates (TNR) were obtained from a 5 × cross validation. The TPR ranged between 87.9–100% whilst the TNR ranged between 0–12.1% (Fig. 4a). Additionally, the receiver operating characteristic (ROC) curves had an area under the curve between 0.99–1.00 (Fig. 4b–f).

### Microglia density differences

**Figure 5:**
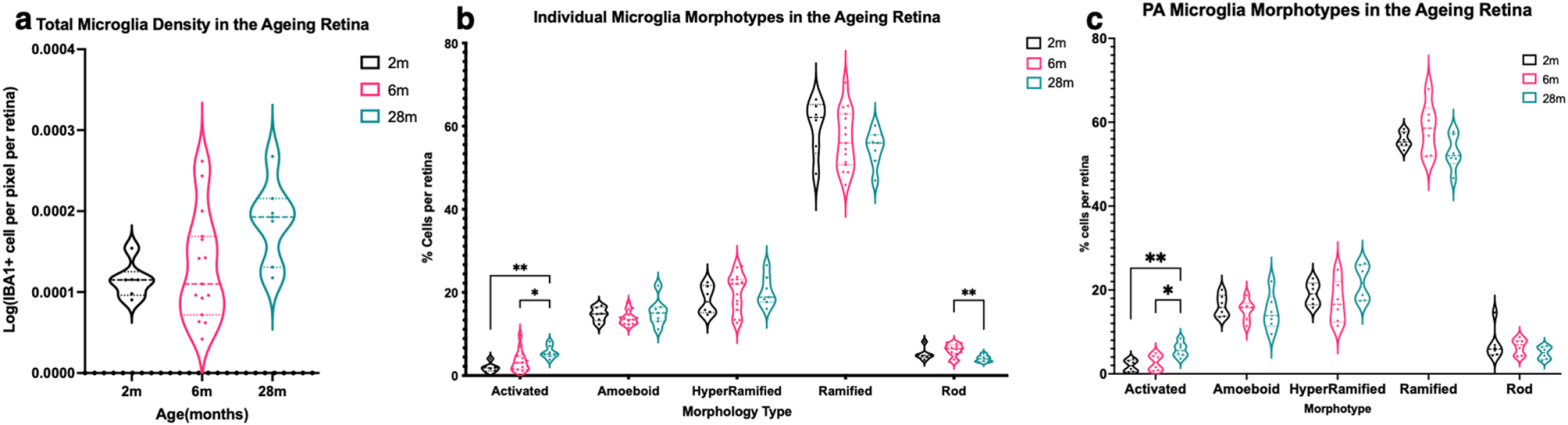
Retinal Microglia density graphs. (**a**) shows a graph of the total microglia density per pixel per retina with ageing. There were no significant differences between each age group (Kruskal Wallis). (**b**) shows a graph of the individual microglia morphotypes in the ageing retina. These were expressed as the percentage of each morphotype cells per retina. There were significant differences in the activated and rod microglia. (**c**) A graph of the PA microglia morphotypes in the ageing retina with the percentage of cells per retina for each morphotype. There were significant differences in the PA activated microglia with increasing age. (**p < 0.01, *p < 0.05, 2-way ANOVA with multiple comparisons). Black = 2 m; Pink = 6 m; Blue = 28 m.

Analysis of the total microglia density of 2 m, 6 m and 28 m retinas showed no significant differences (Fig. 5a). Analysis of the individual microglia morphotypes showed that the percentage of activated microglia per retina increase significantly with age (Fig. 5b). Specifically, there was a significant difference between 2 and 28 m (p < 0.01) and also between 6 and 28 m retinas (p < 0.05) (Fig. 5b). Additionally, the percentage of rod microglia was significantly higher in 6 m retinas compared to that of 28 m (p < 0.01) (Fig. 5b).

### Perivascular area (PA) microglia morphotype density differences

Analysis of the individual PA microglia morphotypes of 2 m, 6 m and 28 m retinas showed a similar trend with that of the activated microglia in the whole retina (Fig. 5b,c). There was a significant difference between 2 and 28 m (p < 0.01) and also between 6 and 28 m retinas (p < 0.05) (Fig. 5c). When the percentage of perivascular area (PA) and non-perivascular area (nPA) activated microglia were compared, there were no significant differences at all age groups.

## Discussion

In this study, we have developed effective retinal image processing and retinal microglia morphotype classification algorithms with a good degree of accuracy. Our ROC results have indicated high discriminative power. Whilst there has been an increase in interest to utilise automated methods to analyse microglia, the present study is the first to successfully investigate automated classification of retinal microglia. For instance, some studies have found effective methods to image and quantify retinal microglia whilst others developed algorithms to extract numerous spatial parameters to maximise data obtained from images of the retina^16,37,38^. Like the retina, similar investigations have been performed using the brain^39^ however, recently, Leyh et al*.,* reported a sophisticated deep learning approach to morphologically phenotype hippocampal and cortical microglia^40^. In this case, a convolutional neural network (CNN) based approach was used to classify ramified, activated, rod-like and amoeboid microglia in mice modelled for ischemic stroke^40^. Although a manually identified training dataset was utilised to develop their CNN, a successful model was obtained with accuracy ranges between 91.67–97.78%^40^. Whilst some sources state that CNN models may achieve better performance accuracies than SVMs^41^, our results (87.9–100%) indicate the capabilities of supervised SVM models. Additionally, our model is capable of analysing the whole retina whilst Leyh et al*.,* used manually obtained sections of the brain^40^.

Using our automated SVM-C, we found that as age increases, the percentage of activated microglia per retina also increased. Increase of activated retinal microglia with ageing or ‘senescent’ microglial activation has previously been reported although these investigations did not use automated machine learning algorithms^8,19,20^. For instance, Damani et al*.,* found shorter and fewer retinal microglia processes in the aged (18–24 months) compared to younger (2-3 months) samples, indicative of the activated morphotype^19^. Another investigation found that in older mice (15 months), the cell body area and number of vertical processes of their retinal microglia had significantly increased compared to the younger mice (4 months)^20^. It was also found that there was a higher expression of P2RY12, the resident microglia marker, in the younger group compared to the older whilst there was a higher expression of CD68, the activation marker, in the older group compared to the younger^20^. This senescent activation has been associated with influencing age-related retinal and neurodegenerative disorders^8,19,20,42^. For instance, Ma et al*.,* observed microglial activation in ageing, could result in the decrease of tight junction proteins (ZO-1, claudin-1) and the increase of proinflammatory cytokines (IL-1β, TNF-α) and factors like vascular endothelial growth factor (VEGF), causing cell structure disorganisation and choroidal neovascularisation (CNV)^43^. These findings, especially CNV, were associated with age-related macular degeneration (AMD)—one of the most prevalent causes of loss of vision in the elderly age group^43,44^. Age is one of the main risk factors of neurodegenerative diseases such as Alzheimer’s disease (AD)^45^. Some findings have shown that microglia in the ageing brain exhibit changes that are also seen in that of AD^45^. These observations resemble both morphological and transcriptional features of activated microglia^45^. Additionally, AD patient brain investigations have revealed that activated microglia reside near amyloid beta plaques^17^. Recently, Salobrar-Gracía et al*.,* found that AD modelled transgenic mice (3xTg-AD), had retinal microglia with shorter and thicker processes and an enlarged cell body area, resembling activated microglia^17^. Together these findings may suggest that senescent changes in the microglia could contribute towards AD pathology. However, to date, there have not been any reports that investigate the profile of microglia with ageing in parallel to AD.

Rod microglia have also been implicated with ageing and age-associated diseases^8,13,14,15,30,46^. From our findings, there is suggestion of their initial role in the retina during an earlier phase of ageing as evidenced by their significantly higher percentage in the 6 m age compared to 28 m. Although there are no investigations on the effect of retinal rod microglia with ageing, Yuan et al*.,* investigated rod microglia in the retina of Sprague–Dawley rats with optic nerve transection (ONT) surgery, a model of the progressive degeneration and loss of retinal ganglion cells, which is a prominent feature of ageing^13,47^. Retinal rod microglia were visible within 1 week of ONT, peaking at 2–3 weeks and diminished after the 6th week^13^. This suggests that rod microglia may be actively involved in the progressive loss of RGCs. Our findings may also shed light on a suggested hypothesis mentioned in literature^4,8,22,48^, where the rod morphotype may be considered one of the intermediate ‘transitioning’ phase within the morphological transformations of microglia. For instance, Tam and Ma found many rod microglia along scratches made on poly-d-lysine and laminin-coated microglia culture chambers^48^. When these cultures had lipopolysaccharide added as a model of further injury, the rod microglia were transformed into amoeboid within 30 minutes^48^. In parallel to morphological observations, the expression of proinflammatory markers such as IL-1ß and TNF were found to be significantly upregulated in the amoeboid microglia seen at 30 min compared to the rod type seen at baseline^48^. As a result, Tam and Ma conclude that rod microglia may not play a direct role in the neuroinflammatory responses to injury^48^. Whilst this may not be directly relatable to the circumstances of ageing, the senescent immunomodulatory effects on the retina may be more extreme at 28 m compared to 6 m. The rod microglia at 6m may have also transitioned into some amoeboid by 28m rather than there being a proliferation of amoeboid cells. This is because there was no change in overall microglia density between 6 m and 28 m whilst there was an increase in the percentage of amoeboid microglia 28 m compared to 6 m.

Our findings show no difference in the overall retinal microglia density and this has also been observed recently although only in the outer segment of the retina^20^. This may further support that retinal microglia may transition between morphotypes^4,8,22,48^ as opposed to undergoing significant levels of proliferation with ageing. For instance, both retinal and brain microglia investigations have reported the longevity of microglia, whereby those in the brain may last for more than 20 years^49,50^. Interestingly, most existing evidence on retinal microglia densities have reported significant increases with increasing age^17,19,20,51,52^. This has been hypothesised to be a compensatory response to senescent microglia becoming dysfunctional^51,52^. However, in every case, these results were obtained from analysis of sections of the retina which may have also been manually analysed^17,19,20,51,52^.

In addition to whole retinal observations, we also investigated retinal microglia in the PA and nPA. The percentage of activated microglia in the PA region also had a significant increase with age whilst there were also no significant differences between PA and nPA activated microglia percentages for each age group. This suggests that there may not be a PA specific change in the population of activated microglia with ageing. Conversely, when Endo et al*.,* investigated retinal microglia morphologies in the vascular or avascular areas, they concluded that there were a greater number of amoeboid cells in the avascular region^53^. This difference may have been due to different age and strain of specimen (postnatal day 5, Slc:ICR mice)^53^. However, their conclusion was drawn from morphological statistics showing that the ‘average process length’ of vascular microglia was greater than the avascular whilst showing no significant difference of the process length per cell or number of processes per cell in the vascular and avascular regions^53^. Additionally, the methods on how each vascular or avascular sections were chosen were not specified^53^. Although the current PA analysis methods may be further automated, it is arguable that this may be a more representative and accurate way to investigate retinal PA microglia morphotyping.

With respect to the technical discussion, these findings have emphasised the difference that may result from using the whole retina to analyse compared to a sectional retina analysis. The current method may be advantageous as it offers a representative, quick and automated way to characterise retinal microglia morphotypes. Future investigations may also observe the expression of specific markers such as P2RY12 and CD68 which are associated with surveillant and phagocytic functions (respectively) to further elucidate the functional associations of these morphological characteristics^20^. Finally, when investigating characteristics of microglia, the influence of microbiomes should be considered. Not only age-related neurodegenerative diseases but also retinal diseases such as AMD are now being associated with microglial pathologies that may be influenced by changes in the gut microbiome^54–56^. Additionally, a plethora of environmental factors such as diet and geographic location may influence this gut microbiome^57^. From this, it can be deduced that comparing even pre-clinical investigations from different geographic locations may unsurprisingly result in contradictions. This may be an unavoidable feature of the currently available worldly research. Therefore, deducing conclusions based on stratified populations of data may be advantageous.

The SVM developed in the present paper is the first of its kind to analyse such large quantities of microglia (entire retinas) with such morphological detail. The ability to process the entire retina not only streamlines research activity but enables a fuller picture of the true distribution of microglia to be gleaned. The ROC curves indicate high discriminating power of this SVM-C, and our results suggest good accuracy—on par with similar machine learning algorithms developed for analysis of microglia in brain sections. The rapid and accurate assessment of microglial information is essential for the high data-throughput requirements of whole-retina analyses, which maximise the utility extracted from each animal.

Our findings relating to an increase in the prevalence of activated microglia in senescence are in line with previous studies which derived their conclusions from simpler data (such as analysis of process length). It is thought that this alteration to microglial morphological makeup is intrinsic to age and disease related microglial changes in the CNS. Regarding the prevalence of rod microglia, some evidence was found to support their presence early in the ageing process (significantly higher prevalence at 6 months compared to other time points). It may be the case that these rod microglia are a transition state towards the amoeboid state, which showed a non-significant trend of increasing in prevalence at the 28-month timepoint.

Our finding of stable microglial densities throughout ageing supports the idea of microglia transitioning between morphologies as opposed to the selective proliferation of these cells. This is in contrast with much existing literature, which states that retinal microglial densities do increase with age, however these findings are notably from analyses which considered only sections of the retina, rather than whole-mount imaging. It is possible that a systematic spatial migration of microglia in ageing is biasing such studies, an effect that would not affect our newly developed whole retinal analysis SVM.

Moving forward, our SVM would be well suited to investigate the relationship between microglial activity markers and microglia morphology, to better understand the nature of the link between the structure and function of these cells. Furthermore, the high level of fidelity in the data acquired by our SVM allows us to detect subtle morphological and functional changes in microglial behaviour across the entire retina. It has been hypothesised that factors beyond the CNS (such as the gut microbiome) can impose drastic alterations on microglial behaviour. Better understanding of this relationship may yield new insights into disease processes, as well as providing critical guidance for the development of pre-clinical experiments targeting microglia.

## Methods

### Animals

Male C57BL/6 mice were bred in the Hellenic Pasteur Institute (HPI, Greece) and University College London (UCL, UK) with all experiments approved by the Institutional Protocol Evaluation Committee (#3492) and the UK Home Office (respectively). The procedures conformed with the ‘Statement for the Use of Animals in Ophthalmic and Vision Research’ (ARVO) following the Animal Scientific Procedures Act 1986 and the study was carried out in compliance with the ARRIVE guidelines. Mice were kept at a 12 h/12 h light/dark cycle with standard mouse chow and water ad libitum. Mice were sacrificed at 2 months (m) (n = 6) (HPI or UCL), 6 m (n = 15) (UCL) or 28 m (n = 7) (HPI) by CO_2_ asphyxiation (HPI) or after anaesthetizing with Ketamine (0.2 ml/25 g) and administration of Euthasol (0.1 ml/10 g) (UCL). They were then perfused with phosphate buffered saline (PBS) and 4% paraformaldehyde (PFA).

### Enucleation and retinal dissection

Perfusion fixed eyes were enucleated then post-fixed at 4 °C overnight (UCL) or post-fixed then incubated in 15% sucrose for one night then in 30% sucrose for another night (HPI). Eyeballs were then dissected, using dissection scissors to make a circumferential cut around the cornea and remove the anterior section. The lens and vitreous were removed, then the retina was gently extracted from the choroid.

### Immunohistochemistry of retinas

Dissected retinas were washed in a mixture of PBS and 0.5% Triton X-100 (Sigma-Aldrich, UK) while placed on a rocker (42 rpm at room temperature). Retinas were then frozen at − 80 °C for 15 min then thawed until completely melted. Afterwards, they were then washed again using PBS and 0.5% Triton X-100. Permeabilised retinas were incubated in a blocking serum solution made of normal donkey (5%) and goat (5%) serums (Sigma-Aldrich, UK), and phosphate buffer (PB, 0.1 M) to minimise non-specific antibody binding. Samples were then stained for microglia and retinal ganglion cells (RGCs) using ionised calcium binding adaptor molecule 1 (Iba-1, the most commonly used marker of microglia; Wako, rabbit anti-Iba1) and RNA binding protein with multiple splicing (RBPMS; Merck, guinea pig anti-RBPMS) respectively. Both the primary antibodies were diluted at 1:1000 using 0.1% bovine serum albumin (BSA, made with 0.1 M PB). These were incubated at room temperature for two days. The samples were then washed in PBS and 0.5% Triton X-100 then incubated at room temperature for 1.5 h with the secondary antibodies consisting of donkey anti-rabbit (555 nm, Alexa Fluor Invitrogen™) and goat anti-guinea pig (647 nm, Alexa Fluor Invitrogen™), both at 1:250 dilution using 0.1% BSA. They were then washed in PB (10 mM). All samples were stained using a randomised complete block design whereby each staining batch included at least 1 retina from every age group. This was to minimise the effects of human errors affecting the staining quality for e.g., a whole experimental group. Each of the stained retinas were carefully removed from the staining well plate, incisions were made to create 4 petals, and placed onto a glass microscopy slide (Merck, UK) ensuring that the curved edges were facing upwards. Mounting solution (Mowiol, Sigma-Aldrich, UK) was applied on the coverslip (Merck, UK) and placed over the retina. Slides were then imaged using a fluorescent microscope (Olympus BX40, Windsor, UK) using a 10 × Olympus lens where 1 pixel = 0.636 μm. Iba-1 and RBPMS were imaged with filters of 555 nm and 647 nm respectively. Z-stack images were obtained using a motorised stage (Prior XYZ joystick, UK) to establish the maximum and minimum limits at which microglia (or RGCs) could be visualised. The integrated imaging software (Image Pro Plus v7, UK) was used to apply the live extended depth of field (EDF) function which allowed high-resolution imaging of RGCs and especially microglia which typically exist in two different layers of the retina that cannot be captured within a single plane of focus. Additionally, the automated stage allows for stitching of tile scan pictures to produce single whole mount image encompassing the entire retina.

### Image processing and automated cell counting

**Figure 6:**
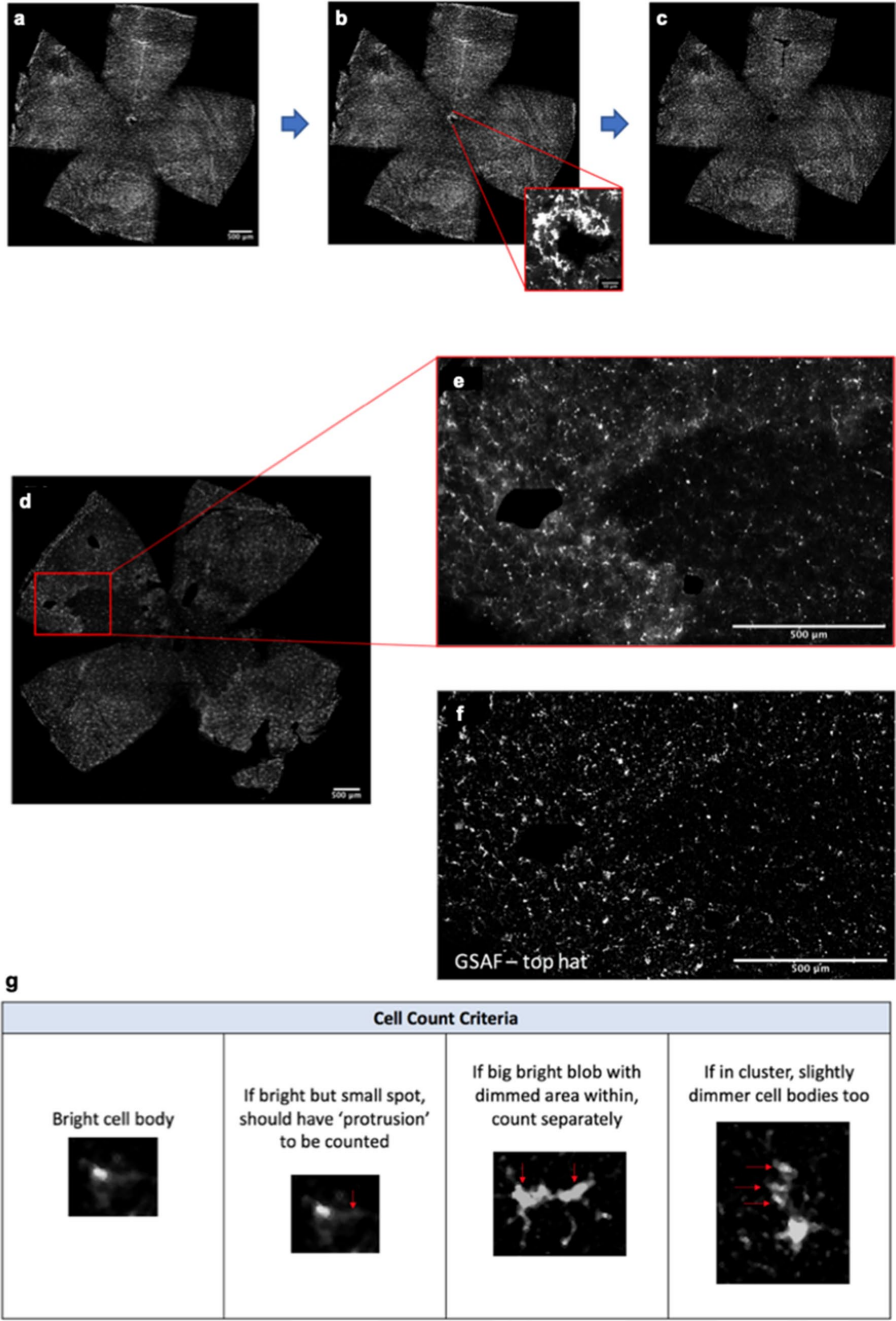
CBCS Development process. (**a**–**c**) shows the artefact removal process where (**a**) shows regions of oversaturated staining especially near the optic nerve head (**b**) and the edges of the retina. (**c**) shows artefacts having been removed to prevent skewing of results due to the oversaturated areas. (**d**) shows the original image with a zoomed section (**e**) showing uneven staining. This was greatly improved by the GSAF function with top hat as seen in (**f**). (**g**) shows the cell count criteria that were utilised to ensure for a consistent counting method between each observer. Scale bars represent 500 μm in (**a**,**d**,**e**,**f**) and 50 μm in (**b**). *GSAF* Gray Scale Attribute Filtering.

All images underwent a standardised artefact removal process using ImageJ (NIH, USA) where oversaturated areas, especially around the optic nerve head (Fig. 6a,b) and the outer edges of the retina, were removed (Fig. 6c). We developed an automated cell body counting script (CBCS) (Supplementary Fig. S1 online). Briefly, the CBCS duplicated the original image for the purpose of overlays at the end, altered the gamma, applied ‘Despeckle’ and ‘Remove Outliers’ to remove noise, then converted the fluorescent image to greyscale and utilised the ‘Gray Scaler Attribute Filtering’ function to enhance the microglia bodies and equalise the uneven background (Fig. 6d–f). Through the development and optimisation of the CBCS, two types of ‘Auto Local Threshold’ were examined. This was done by comparing the number of cells, from sections of the retina (500 µm × 500 µm, n = 30), deduced from manual counts (obtained from three observers), with those deduced from two preliminary versions of the CBCS, one which used the ‘Moments’ thresholding or the other, the ‘IsoData’ thresholding. Manual counts were performed following the criteria in Fig. 6g to maintain consistency across the observers, in what was defined as a cell. The CBCS uses the ‘Analyse Particles’ function which analyses up to 34 morphological and spatial parameters of the counted cell bodies including the x and y co-ordinates, area, solidity, Feret’s maximum (${F}_{max}$), Feret's minimum (${F}_{min}$) and more as mentioned in literature^57^. Finally, the CBCS allows for these automatically recognised cell bodies to be visualised with each cell shown as an overlay on top of the original image.

### SVM-C development and validation

An SVM is a supervised machine learning algorithm which is trained using model data for each class to be able to then classify testing data into individual classes. To develop the training data, a criterion, defining the cell body characteristics of each morphotype, consisting of qualitative and quantitative descriptors, was established. The qualitative descriptors were found by performing a literature search using words including: ‘ramified’, ‘amoeboid’, ‘rod’, ‘activated’, ‘hyper-ramified’, ‘microglia’, ‘retina’ and ‘cell body’. A similar method was applied to identify already-identified quantitative descriptors of each morphotype body parameters. However, to our knowledge, there was no existing literature evidence which compared morphometric parameters between the cell bodies of each of the morphotypes of retinal microglia. Therefore, an iteratively developed semi-subjective approach was used to identify further quantitative descriptors that distinguish the different morphotypes in the following way.

Firstly, the co-ordinates output by the CBCS were used to develop an auto segmentation script (AS) (Supplementary Fig. S2 online) (MATLAB, R2020a) which produced segmentations of binarized images (ASBI) (Fig. 2c), from the whole retina that only showed the central ‘blob’ i.e., the counted cell body (without any visibility from parts of adjacent cell bodies). The AS was run on randomly chosen whole-mounted retinal images (random number generator, n = 1 per group). Secondly, the qualitative descriptors were used as a criterion to categorise ASBIs to the individual morphotypes. In order to confirm that the ASBIs were categorised into the correct morphotypes, their cell body co-ordinates obtained from the CBCS were then used to track back to the original non-binarised histology image. This allowed visualisation of correct morphotype categorisation by examining the cell process characteristics which also play a major role in distinguishing each morphotype. Then thirdly, the CBCS output parameters including the feret’s distances and solidity were examined to establish those those that would allow easier distinction between morphotypes for the semi-subjective process to gather the training data for the SVM. For example, using area as a parameter to distinguish between a rod-like shape from one that is ramified-like may be more difficult than using the ${F}_{max}$ , the greatest length between two tangents that are parallel on an object (Fig. 2d). Based on the qualitative descriptors established from literature evidence, the cell body of ramified microglia have been described to have the smallest ${F}_{max}$, followed by that of amoeboid microglia (Fig. 2c). The ${F}_{max}$ of the other morphotypes’ cell bodies have also been described as being similar or larger. As a result, the ${F}_{max}$ of amoeboid cell bodies was examined to deduce a limit that could quantitatively distinguish ramified cell bodies from the other non-ramified types. Due to the lack of existing retinal data, the minimum ${F}_{max}$ limit of amoeboid cell body was calculated as the average value from the results from a previous investigation which reported comparative analysis of brain microglia cell body parameters between individual morphotypes and the average ${F}_{max}$ of amoeboid cell bodies deduced from the CBCS data of the amoeboid categorised ASBI images (following the processed explained so far)^10^. Of the non-ramified morphotypes, the rod and hyper-ramified types were considered to have more similar features (especially qualitative descriptors (Fig. 2c). Therefore, the CBCS parameters were further observed to identify parameters that would distinguish the two morphotypes. This included the solidity ($\frac{Area}{Convex Area}$) and the Feret’s diameter ratio (FDR) (calculated by $\frac{{F}_{max}}{{F}_{min}}$)**.** As a result, the ${F}_{max}$ and FDR were included as the quantitative descriptions as part of the morphotype defining criterion. The training data were compiled using this morphotype defining criterion and by cross-referencing with the ASBIs (n = 240 cells per morphotype, n = 1200 in total). Although these cells were obtained with partial manual influence, the data for each of the 34 parameters (explained in Supplementary Table S1 online) for each of the 1200 cells were included in the training dataset.

**Figure 7:**
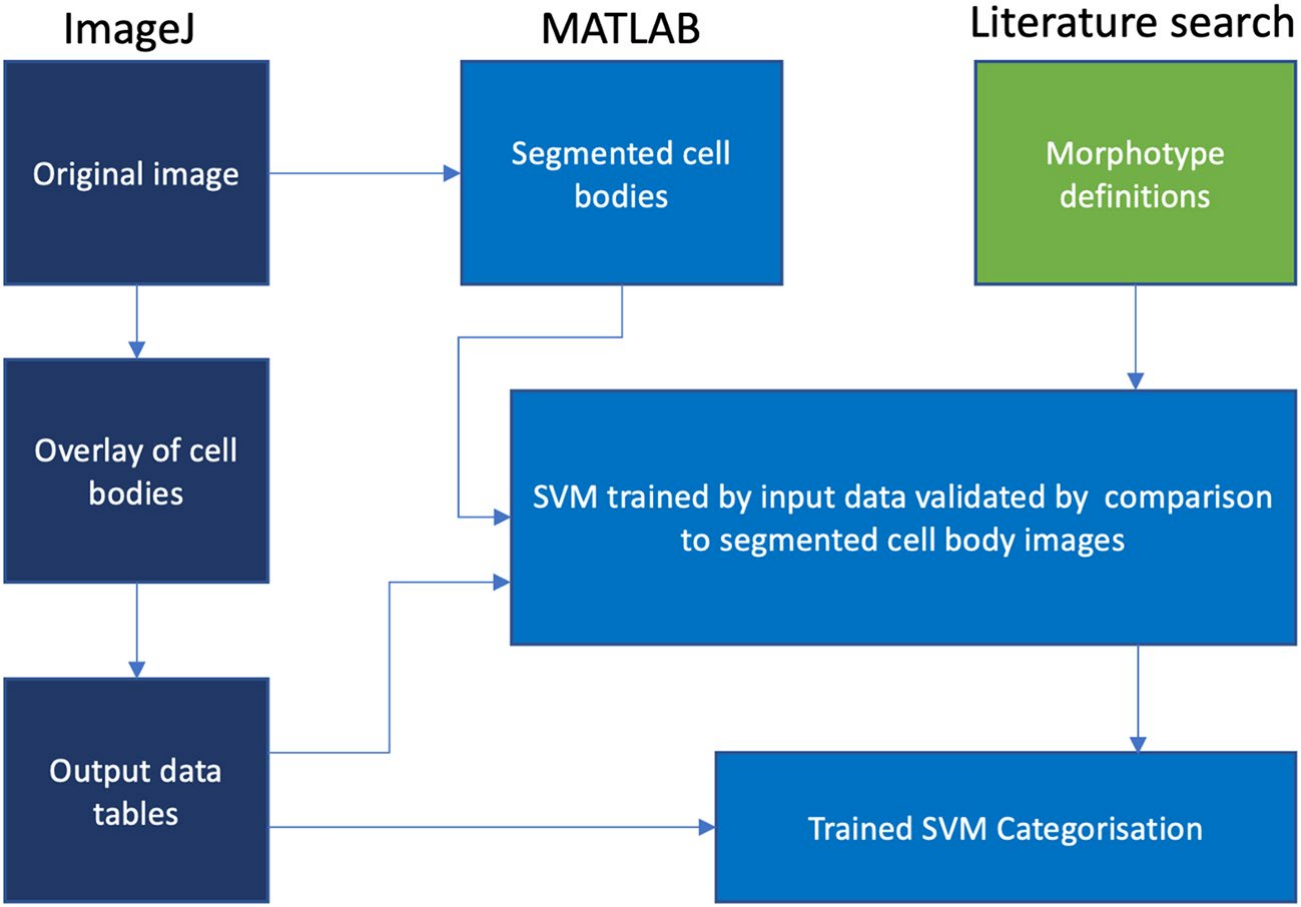
SVM-C Development process. The main processes involved in developing the SVM-C. Dark blue are processes performed using ImageJ, lighter blue using MATLAB, and green using literature search.

A linear SVM model was selected (MATLAB Classification Learner Application, R2020a). This type of model could automatically sort through the data on the parameters within the training data for each morphotype and establish the optimal hyperplanes that separate the data between each morphotype. This nature of linear SVMs made it ideal to compensate for the semi-subjective selection method of the training dataset. The training data were imported and a five-fold cross-validation was applied. The data were divided into 5 groups, among them, 4 groups were used to train and then 1 group to test the machine and repeated five times to calculate the average accuracies and test error rates. These were used to produce the receiver operating characteristic (ROC) curves and confusion matrices of the resultant SVM-Classifier (SVM-C) model which was then used to classify the morphotypes of each counted cell identified by the CBCS (Supplementary Fig. S3 online). This process has been represented in the flow chart in Fig. 7.

### Perivascular area (PA) analysis

**Figure 8:**
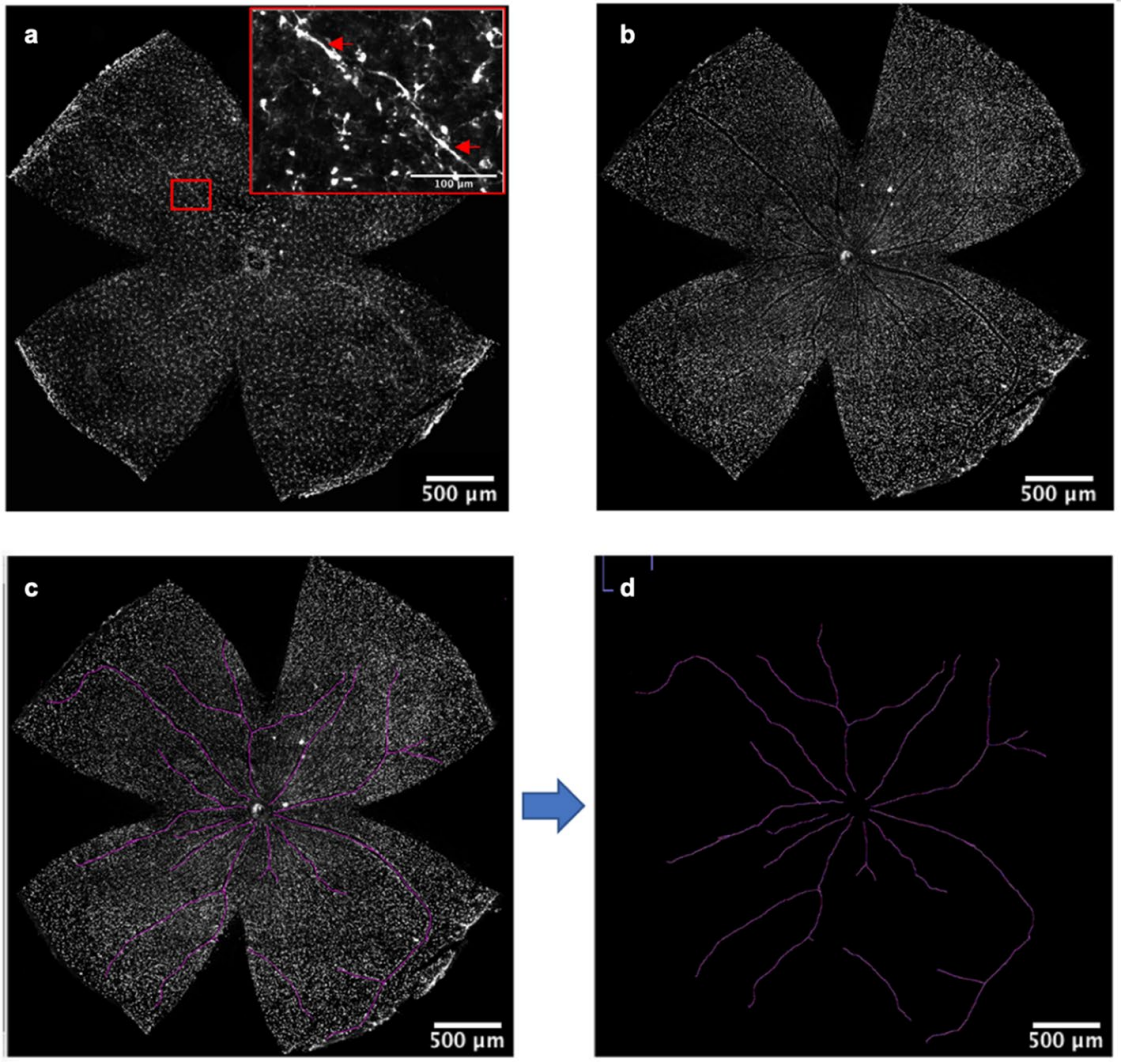
Perivascular Area Analysis Process. (**a**) and (**b**) display the same sample imaged in the Iba-1 (**a**) or RBPMS (**b**) channel. The vessel may be distinguishable in (**a**) as shown by the enlarged insert and red arrows. (**b**) shows that the vessel is more clearly visible. (**c**) shows the retinal vasculature having been traced onto the image. (**d**) shows an image of just the retinal vasculature. Scale bars represent 500 μm in all images whilst the enlarged section in (**a**) shows 100 μm.

The ‘Overlay Brush’ (ImageJ)^58^ was used to trace the vessels on the RBPMS stained images which had a clearer visualisation of retinal vessels (Fig. 8a vs b,c) (n = 6 per group). An image containing only the vessels was then obtained by selecting ‘Process > Math > Subtract… > value = 1000’ (Fig. 8d). Then a perivascular area analysis (PAA) script (Supplementary Fig. S4 online) was developed and applied to investigate the PA morphotypes (MATLAB, R2020a). The PAA binarizes the vessel-only image by allocating non-black pixels as ‘1’ and black pixels as ‘0’ and any pixel that was $\le$ 59.5 pixels from a non-black ‘1’ pixel was regarded as PA. Any PA that was co-localised with the co-ordinates of any counted cell from the CBCS output was classified as a PA microglia.

### Statistical analysis

The resulting data from each retina were expressed as a percentage of the whole retina. This was used to accommodate the difference in area of retina analysed per sample. The cell solidity, FDR, total and individual microglia morphotype density and PA morphotype data were analysed using GraphPad Prism 9 (GraphPad Software). P-values of < 0.05 were considered significant.

## Supplementary Information

Supplementary Information.

## Data Availability

All code used for this project is available in the supplementary figures S1-4. The SVM-C model used in our classification can be provided upon request.
