# Supplementary material for: Automated characterisation of microglia in ageing mice using image processing and supervised machine learning algorithms
Source: Sci Rep. 2022 Feb 2;12:1806. doi: 10.1038/s41598-022-05815-6 (PMC8810899; doi:10.1038/s41598-022-05815-6)
Supplement: Supplementary file 1 — Supplementary Information. [file 41598_2022_5815_MOESM1_ESM.pdf]

Automated characterisation of microglia in ageing mice using image processing and supervised machine learning algorithms.

Soyoung Choi[1], Daniel Hill[1], Li Guo[1], Richard Nicholas[1,2,3], Dimitrios Papadopoulos[4,5] and Maria Francesca Cordeiro[1,6,*]

[1]UCL Institute of Ophthalmology, London, EC1V 9EL, United Kingdom.
[2]Division of Brain Sciences, Department of Medicine, Imperial College, London, UK.
[3]Population Data Science, Swansea University Medical School, Swansea, SA2 8PP, UK.
[4]Laboratory of Molecular Genetics, Hellenic Pasteur Institute, Athens, Greece, 11521.
[5]School of Medicine, European University Cyprus, Nicosia, 2414, Cyprus.
[6]Imperial College Ophthalmology Research Group, Imperial College London, London, London, United Kingdom.
[*]corresponding. m.cordeiro@ucl.ac.uk

Supplementary Figures

S1: Cell Body Counting Script (CBCS)

ImageJ script for automatic whole retinal image processing and retinal microglia cell body counting

```
run("Duplicate...", "title=Image1.tif");
run("Duplicate...", "title=Image2.tif");
selectWindow("Image1.tif");
run("Gamma...", "value=1");
run("Despeckle");
run("Remove Outliers...", "radius=2 threshold=50 which=Bright");
run("Grays");
run("Gray Scale Attribute Filtering", "operation=[Top Hat] attribute=[Box Diagonal] minimum=100 connectivity=4");
run("Enhance Contrast...", "saturated=1");
run("Duplicate...", "title=Image1.tif")'
run("Auto Threshold", "method=Moments ignore_black ignore_white");
setOption("BlackBackground", false);
run("Convert to Mask");
run("Analyze Particles...", "size=35-infinity pixel circularity=0-1.00 show=Outlines display clear summarize add");
selectWindow("Image2.tif");
run("Add Image...", "image=[Drawing of Image1.tif] x=0 y=0 opacity=0");
selectWindow("Image1.tif");
close();
selectWindow("Drawing of Image1.tif");
close();
```

S2: Autosegmentation Script (AS)

MATLAB script for automatically segmenting binarized images of each counted microglia cell body

```
clc;
clear all;
close all;
circle=1;
cellsize=49;
bordersize=cellsize*3;

mask=ones(cellsize*2,cellsize*2,'uint8');

if circle == 1
    for r = 1:cellsize*2
        for c = 1:cellsize*2
            if round(sqrt((abs(c-cellsize))^2 + (abs(r-cellsize))^2)) > cellsize
                mask(r,c)=0;
            end
        end
    end
end

thisfilename=mfilename('fullpath');
location=which ([thisfilename '.m']);
[save_dir,~,~] = fileparts(location);
```

```
countfiles=dir([save_dir '/count/*.xlsx']);
countfilenames= {countfiles.name};

imagefiles=dir([save_dir '/image/*.tif']);
imagefilenames= {imagefiles.name};

for multi = 1:length(countfiles)

[countdata,~,~]= xlsread([[save_dir '/count/'] countfilenames{multi}]);

disp('excel read done')


im=imread([[save_dir '/image/'] imagefilenames{multi}]);
disp('image read done')
res=size(im);

cropim=zeros(cellsize*2,cellsize*2,length(countdata)-4,'uint8');

countdata(:,9:10)=countdata(:,9:10)+(bordersize/100);

image=zeros(res(1)+(bordersize*2),res(2)+(bordersize*2),'uint8');
image(bordersize+1:res(1)+bordersize,bordersize+1:res(2)+bordersize)=im;

mkdir([save_dir '/COutput/' imagefilenames{multi}])

for cellnum = 1:length(countdata)

Ty=round(countdata(cellnum, 10)*100)-cellsize;
Lx=round(countdata(cellnum, 9)*100)-cellsize;
By=round(countdata(cellnum, 10)*100)+cellsize;
Rx=round(countdata(cellnum, 9)*100)+cellsize;

cropim(:,:,cellnum)=image(Ty+1:By,Lx+1:Rx).*mask;

im3 = imbinarize(cropim(:,:,cellnum))

labeledImage = bwlabel(im3)

measurements = regionprops(labeledImage,'Centroid');
centroids = [measurements.Centroid];
xCentroids = centroids(1:2:end);
yCentroids = centroids(2:2:end);

distances = sqrt((49-xCentroids).^2 + (49-yCentroids).^2);

[minDistance, indexOfMin] = min(distances);

centralBlob (:,:,cellnum) = labeledImage == indexOfMin;

imwrite(centralBlob (:,:,cellnum),[save_dir '/COutput/' imagefilenames{multi} '/' num2str(cellnum) '.tif'])
end

disp(['done ' num2str(multi) ' of ' num2str(length(countfiles))])
end
```

S3: Support Vector Machine Classifier Model Application Script (SVM-C)

MATLAB script which automatically predicts the morphotype of each counted cell based on the *svmtrainedmodel2.mat* model. The model was created using training data obtained using methods described in *Methods*: *SVM-C development and validation*. Model may be provided upon request

```
clear all

thisfilename=mfilename('fullpath');
location=which ([thisfilename '.m']);
[save_dir,~,~] = fileparts(location);

isthismac = 1
load svmtrainedmodel2.mat

yfit = trainedModel1.predictFcn(countResults35)
```

```
 if isthismac==1
    CS = cellstr(yfit)
     makeatable= array2table(CS);
     writetable (makeatable, [save_dir 'Sample.xlsx']);

    else

    outputcell{1}='morphology';


   out={[outputcell; num2cell(mat)]};


         xlswrite([name 'table.xlsx'], out{1})
    end
```

S4: Perivascular Area Analysis Script (PAA)

MATLAB script which automatically distinguishes PA retinal microglia by comparing the vessel location with the co-ordinates of each counted retinal microglia cell

```
clc;
clear all;
close all;

A=imread('SampleVessel.jpg');

b=im2bw(A,0);

disp ('binarise image complete');

z=bwdist(b);

disp ('bwdist finished');

thisfilename=mfilename('fullpath');
location=which ([thisfilename '.m']);
[save_dir,~,~] = fileparts(location);

files=dir([save_dir '/SamplecountResults.xlsx']);
filenames= {files.name};
data=cell2mat(filenames);
[num,~,~]= xlsread([save_dir '/' data]);

disp ('xlsread finished');

num(:,10)=round(num(:,10)*100);
num(:,9)=round(num(:,9)*100);

for j=1:length(num);

        if z(num(j,10), num(j,9))<59.5;

                num(j,38)=1;

         else, num(j,38)=0;

    end;

end;

xlswrite(data, num(1:length(num),38));
```

Supplementary Table

S1: Parameters outputted by the 'Analyse Particles' function

Parameter measurements in relation to the retinal microglia cell bodies are summarized below based on the descriptions mentioned in [1].

Summarised Parameters	Description
Area	The total area of the counted microglial cell body given in square units
Mean gray value	The total of the gray values from all pixels in the counted microglial cell body divided by the total number of pixels in the counted cell body
Standard deviation	The standard deviation of the gray values used to obtain the mean gray value of the counted microglial cell body
Modal gray value	The most common gray value of the microglial cell body
Min&Max gray level	The minimum and maximum gray value of the microglial cell body
Centroid	The x and y co-ordinates of the central point of the counted microglial cell body (shown as X and Y)
Center of mass	The brightest mean of the x and y co-ordinates of all the pixels of the counted microglial cell body (shown as XM and YM)
Perimeter	The distance around the boundary of the counted microglial cell body
Bounding rectangle	The smallest rectangle that can surround the counted microglial cell body where the top left corner co-ordinates are shown as BX and BY (also shows width and height)
Fit ellipse	The counted microglial cell body is fitted with an ellipse around it. The primary axis of the ellipse is expressed as Major, the secondary as Minor. The angle between the primary axis and the X-axis is expressed as the Angle.
Circularity	The closer to a perfect circle the microglial cell body appears, the closer to 1.0 this value is. As a shape becomes elongated, the value approaches 0.0. ($Circularity = 4\pi \frac{Area}{Perimeter^2}$)
Aspect ratio	The ratio of the Major and Minor axis ($Aspect\ ratio = \frac{Major\ axis}{Minor\ axis}$)
Roundness	The inverse of the aspect ratio or $Roundness = 4\frac{Area}{\pi \times Perimeter^2}$
Solidity	The solidity of a counted microglial cell body can be expressed as $Solidity = \frac{Area}{Convex\ area}$
Feret's diameter	The greatest length between two tangents that are parallel on the counted microglial cell body is expressed as the Feret (Feret's maximum). The starting co-ordinates of the Feret are shown as FeretX and FeretY. The shortest length between two tangents that are parallel on the counted cell body is the MinFeret (Feret's minimum). The angle of the Feret's diameter is shown as FeretAngle.
Integrated density	The total of the values of pixels within the counted cell body is shown as RawIntDen
Median	The median value of the pixels in the counted microglial cell body
Skewness	The measure of how skewed the distribution of pixel values are of the counted microglial cell body (described as the '3rd order moment about the mean').
Kurtosis	The measure of height and sharpness of the central peak (described as the '4th order moment about the mean')
Area fraction	The fraction of pixels within the thresholded image of the counted microglial cell body that is shown in red (shown as %Area)
Stack position	The stack position, with regards to the slice, channel and frame, of the counted microglial cell body

Reference:

1. T. Ferreira. & Rasband, W. ImageJ User Guide - IJ 1.46r. IJ 1.46r (2012).
